# Wettability Manipulation by Interface-Localized Liquid Dielectrophoresis: Fundamentals and Applications

**DOI:** 10.3390/mi10050329

**Published:** 2019-05-16

**Authors:** Jitesh Barman, Wan Shao, Biao Tang, Dong Yuan, Jan Groenewold, Guofu Zhou

**Affiliations:** 1 Guangdong Provincial Key Laboratory of Optical Information Materials and Technology and Institute of Electronic Paper Displays South China Academy of Advanced Optoelectronics, South China Normal University, Guangzhou 510006, China; jiteshb@m.scnu.edu.cn (J.B.); shaowan@m.scnu.edu.cn (W.S.); jg@denk-werk.nl (J.G.); guofu.zhou@m.scnu.edu.cn (G.Z.); 2 National Center for International Research on Green Optoelectronics, South China Normal University, Guangzhou 510006, China; 3 Van’t Hoff Laboratory for Physical and Colloid Chemistry, Debye Research Institute, Utrecht University, Padualaan 8, 3584 CH Utrecht, The Netherlands; 4 Shenzhen Guohua Optoelectronics Tech. Co. Ltd., Shenzhen 518110, China

**Keywords:** liquid dielectrophoresis, dielectrowetting, wettability manipulation, governing force, microfluidics

## Abstract

Electric field-based smart wetting manipulation is one of the extensively used techniques in modern surface science and engineering, especially in microfluidics and optofluidics applications. Liquid dielectrophoresis (LDEP) is a technique involving the manipulation of dielectric liquid motion via the polarization effect using a non-homogeneous electric field. The LDEP technique was mainly dedicated to the actuation of dielectric and aqueous liquids in microfluidics systems. Recently, a new concept called dielectrowetting was demonstrated by which the wettability of a dielectric liquid droplet can be reversibly manipulated via a highly localized LDEP force at the three-phase contact line of the droplet. Although dielectrowetting is principally very different from electrowetting on dielectrics (EWOD), it has the capability to spread a dielectric droplet into a thin liquid film with the application of sufficiently high voltage, overcoming the contact-angle saturation encountered in EWOD. The strength of dielectrowetting depends on the ratio of the penetration depth of the electric field inside the dielectric liquid and the difference between the dielectric constants of the liquid and its ambient medium. Since the introduction of the dielectrowetting technique, significant progress in the field encompassing various real-life applications was demonstrated in recent decades. In this paper, we review and discuss the governing forces and basic principles of LDEP, the mechanism of interface localization of LDEP for dielectrowetting, related phenomenon, and their recent applications, with an outlook on the future research.

## 1. Introduction

In the last few decades, the wettability of liquids on a solid surface became a very extensively researched topic in surface science due to its fundamental and crucial role in various aspects of domestic life and industry, such as water collection by plant leaves, the self-cleaning effect observed in lotus leaves and feathers of birds, and the paint industry, to name a few [1]. The wettability of a chemically and topographically homogeneous surface is defined by Young’s contact angle via balancing the interfacial forces acting at the three-phase contact point of a droplet in mechanical equilibrium as follows:

(1) $$\cos\theta_{e}=\frac{\gamma_{SV}-\gamma_{SL}}{\gamma_{LV}},$$

where $\gamma_{ij}$s are the different interfacial energies, with i,j representing *S*, *L*, and *V*, which are the abbreviations of the corresponding media as solid, liquid, and vapor, respectively. However, in reality, there exists no ideally homogeneous surface, as every solid surface possesses small inhomogeneity in the form of roughness or chemical impurity or both, which often reflects in the measurement of the contact-angle hysteresis [2], i.e., the difference between the advancing angle and receding angle. For a fixed liquid and a solid surface, the wettability or the contact angle is a constant quantity. However, most applications benefit from the controllability of wetting properties of the liquid and the solid surface involved in the system. The active manipulation of wettability of a liquid on a solid surface through external stimuli, such as electric field, magnetic field, pH, heat treatment, ultraviolet (UV) irradiation, etc., became a modern trend in microfluidics and optofluidics devices [3,4]. Of these external stimuli, the electric field controls the wettability of a liquid through an electrohydrodynamics effect at the three-phase contact line of a droplet via two mechanisms in particular: electrowetting and liquid dielectrophoresis (LDEP) [5].

In the electrowetting mechanism, especially the electrowetting on dielectrics (EWOD) configuration [6,7], the apparent contact angle of a conducting droplet is reversibly modulated by applying a direct current/alternating current (DC/AC) voltage between the droplet and the conducting substrate, separated by a hydrophobic dielectric thin layer. Upon the application of the electric potential across the droplet and the substrate, the free electrical charges (ions) accumulate immediately at the interfaces of the droplets and, as a result, a Coulombic force is developed under the electric field. The charge density becomes maximum at the three-phase contact line with the corresponding maximum outward component of the Coulombic force, which pulls the liquid droplet to spread via the deformation of the air–liquid interface. The principle of EWOD was used extensively in various fields [6,8,9,10,11,12,13]. Unlike EWOD, liquid dielectrophoresis (LDEP) does not need any free charges (ions), but requires polarization. LDEP induces hydrodynamic motion in the liquids with permanent or induced dipoles via the application of a non-homogeneous electric field [14,15,16]. In the past, LDEP was used as a technique to execute various operations to manipulate bulk dielectric liquids, and it evolved as an alternative driving technique for microfluidics [17,18,19,20]. In principle, the LDEP force is a bulk force, whereas the EWOD is an interface-localized mechanism for the manipulation of the wetting of liquid droplets. Recently, McHale et al. first demonstrated the LDEP force as a highly localized force near the three-phase contact line of a droplet using co-planar linear interdigitated electrodes [21]. Due to its similarity with the electrowetting phenomenon, the authors also used the term dielectrowetting, which provides direct control over the wettability of dielectric liquids [21,22,23,24]. The typical range of modulation of the apparent contact angle is higher than that of EWOD. Additionally, the dielectric liquid droplet could be spread into a thin liquid film with wrinkles, overcoming the contact-angle saturation problem in EWOD. This ability generated many exciting applications based on the principle of dielectrowetting [25,26]. The observations in dielectrowetting are apparently similar to those in EWOD; however, fundamentally, it involves the manifestation of the LDEP force highly localized near the three-phase contact line using co-planar interdigitated electrode geometry. 

## 2. Liquid Dielectrophoresis

**Figure 1:**
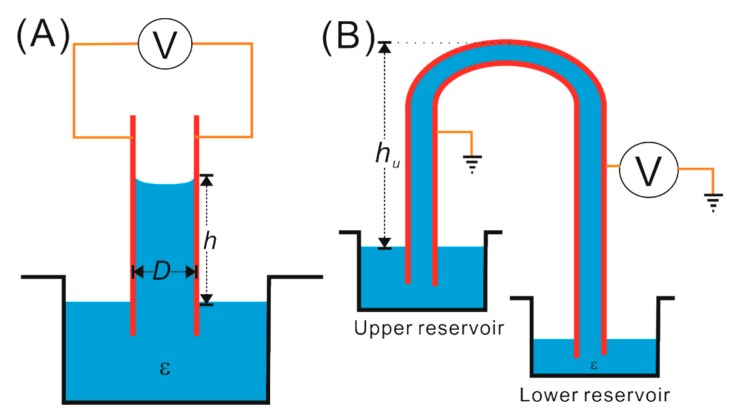
Schematic representation of (**A**) Pellat’s classic experiment of dielectric liquid actuation against gravity by a non-homogeneous electric field, and (**B**) dielectric siphon.

The first experimental demonstration of dielectric liquid (insulating oil) motion by electric field against gravity was reported by Pellat in 1895 [14]. Figure 1A shows the schematic design of Pellat’s classic experiment consisting of two parallel conducting plates, separated by a gap D and partially immersed in a reservoir of dielectric liquid with density ρ and relative permittivity ε. Upon the application of voltage V between the plates, a gradient of electric field is generated at the liquid–air interface, actuating the dipoles inside the liquid to move against the gravity to a new equilibrium height h. Here, the upward motion of dielectric liquid against gravity by the force generated due to the non-homogeneous distribution of fringe electric field intensity is known as the liquid dielectrophoresis (LDEP) effect. By balancing the polarization force acting on the liquid with the gravitational pull on the liquid column, the height rise h can be expressed as follows [16,27]:

(2) $$h\sim \left(\varepsilon -\varepsilon_{0}\right)E^{2}/2\rho g,$$

where E= V/D, g is the acceleration due to gravity, and $\varepsilon_{0}$ is the free space permittivity. However, this exciting phenomenon of dielectric liquid actuation by a non-uniform electric field went overlooked and, hence, remained unexplored by scientists over the next 80 years. In 1971, Jones et al. demonstrated a dielectric siphon based upon the LDEP mechanism, in which they could actuate controlled flow of a dielectric liquid between two reservoirs, kept at different heights by applying a potential difference of 19 kV (see Figure 1B) [15]. Figure 1B shows the schematic of the dielectric siphon made of two inverted U-shaped parallel electrodes separated by a distance s = 3 cm. Once the dielectric liquid is actuated by an electric field to the height of $h_{u}$, the flow of liquid from the upper reservoir to the lower reservoir is established due to the pressure difference between the reservoirs. However, the siphon fails to work if the liquid does not reach to a certain height $h_{u}$. To have a deeper understanding of the LDEP phenomenon, the basic governing electrohydrodynamic forces are analyzed in the section below.

### 2.1. Basic Governing Force Analysis

Dielectrophoresis (DEP), as the name suggests, is a commonly observed phenomenon with dielectrics, involving the translation motion of neutral matter in a non-uniform electric field caused by the polarization effect. The electrohydrodynamic (EHD) motion and the change of the liquid–vapor interface shape of an insulating liquid actuated by the DEP force is known as liquid dielectrophoresis (LDEP). To understand the LDEP effect, we need to analyze the electrostatic interaction with the liquid molecules. In a uniform electric field, the electron cloud of a neutral molecule is slightly displaced toward the positive polarity of the field due to the electrostatic attraction giving rise to the induced dipole moment ($\vec{p}$), which orients opposite to the electric field. The same neutral molecule in a non-uniform electric field ($\vec{E}$), in addition to induction and orientation of dipole moment ($\vec{p}$), will experience an extra net electrostatic force ($\vec{p}\cdot \vec{\nabla }\vec{E}$) toward the direction of higher field intensity. However, a bulk material cannot be approached as a single dipole; instead, the polarization force is calculated by incorporating the bulk polarization density ($\vec{P}$), i.e., the number of dipoles per unit volume. The calculated electrostatic force density with microscopic polarization density ($\vec{P}$) for a bulk material is known as Kelvin polarization force density.

(3) $${\vec{f}}_{KP}=\vec{P}\cdot \left(\vec{\nabla }\cdot \vec{E}\right).$$

A linear dielectric material with susceptibility ($\chi_{e}$) follows the electrostatics relationship for polarization $\vec{P}=\varepsilon_{0}\chi_{e}\vec{E}=\varepsilon_{0}\left(\varepsilon_{r}-1\right)\vec{E}$, where $\varepsilon_{r}$ is the dielectric constant. Therefore, the Kelvin polarization force density for a linear dielectric takes the form as

(4) $${\vec{f}}_{KP}=\frac{1}{2}\varepsilon_{0}\left(\varepsilon_{r}-1\right)\vec{\nabla }\left(\vec{E}\cdot \vec{E}\right).$$

Equation (4) depicts the strength of the force on a dielectric material in ambient air medium that depends on the polarizability, the volume of the attracted medium, and the field intensity squared. 

However, the Kelvin polarization force density only demonstrates the interaction between the dipole moments present in the dielectric material, whether permanent or induced by the electric field. In reality, the liquid could contain free charges in the form of ions and impurity and, upon that, the interaction among the dipoles could lead to a contribution to the exerted force and the electric field. All the effects can be summarized in one equation by writing the electrostatic force density, which originates when an electric field is exposed to an electrically linear and incompressible liquid medium [5].

(5) $${\vec{f}}^{e}=\rho_{e}\vec{E}-\frac{1}{2}{\left|E\right|}^{2}\vec{\nabla }\varepsilon +\vec{\nabla }\left[\frac{1}{2}\left(\varepsilon -\varepsilon_{0}\right){\left|E\right|}^{2}\right],$$

where $\rho_{e}=\vec{\nabla }.\varepsilon \vec{E}$ is the free charge density inside the liquid. The first term in Equation (5) describes the interaction of free charge inside the liquid with the electric field, and the second and third terms together signify the DEP polarization force density, which takes the same form as Kelvin polarization force density in Equation (4) for this special case. On the other hand, the first two terms in Equation (5) represent the Korteweg–Helmholtz force density, which basically accounts for the interfacial force density due to the free charge at the interface and the variation of electrical properties at the interface between two media. 

For the complete electrohydrodynamics (EHD) force, the Navier–Stokes equations governing the hydrodynamics need to be coupled with the electrostatics via deriving the Maxwell stress tensor $T^{M}$ from the electrical force density ${\vec{f}}^{e}$. After re-arranging Equation (5), the electrical force density can be written as

(6) $${\vec{f}}^{e}=\left(\vec{\nabla }\cdot \varepsilon \vec{E}\right)\vec{E}+\left(\varepsilon -\varepsilon_{0}\right)\vec{E}\cdot \vec{\nabla }\vec{E}=\vec{\nabla }\cdot \left(\varepsilon \vec{E}\vec{E}-\frac{1}{2}E^{2}\vec{I}\right)=\vec{\nabla }\cdot {\vec{T}}^{M},$$

where $\vec{I}$ is the unit tensor, and ${\vec{T}}^{M}=\varepsilon \vec{E}\vec{E}-\frac{1}{2}E^{2}\vec{I}$ is the Maxwell stress tensor based on the electrical force density. Therefore, the electrohydrodynamics force on a fluid of volume *V* can be calculated by a simple surface integral of the Maxwell stress tensor over the surface *S* bounding the volume as

(7) $$\vec{F}=\int^{\text{​}}{\vec{f}}^{e}dV=\int^{\text{​}}{\vec{T}}^{M}\cdot \vec{n}ds,$$

where $\vec{n}$ is the unit vector outward normal to the surface element d*s*.

### 2.2. Equivalent Electrical Circuit Model

**Figure 2:**
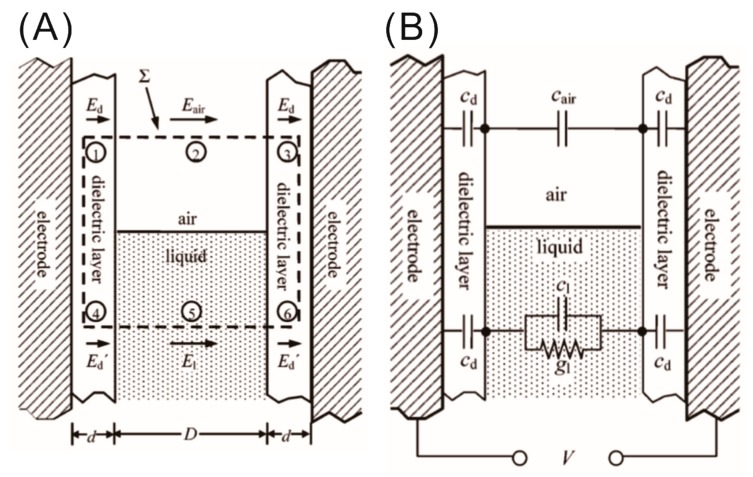
(**A**) Schematic representation of the closed surface integral Σ and corresponding area for calculation of net vertical force of electrical origin on the liquid between insulator-coated electrodes. The choice of closed surface integral is reduced to a simple summation of six discrete areas denoted by 2 for the contribution of air, 5 for the contribution from liquid, and 1 and 3, and 4 and 6 for the contribution from the dielectrics above and below the air–liquid interface, respectively. (**B**) Schematic representation of the equivalent resistor/capacitor (RC) circuit model for determination of the electric field in different regions. Images are reproduced from Reference [30] with the permission of the American Chemical Society.

Jones and his co-workers also demonstrated that, in addition to the actuation of ideally dielectric liquids, the LDEP force can also be applied to actuate aqueous liquids which are partially conducting or leaky dielectric [28,29,30]. They studied the actuation of leaky dielectric liquids between two parallel plates with a thin insulator coating by applying an AC electric field using a lumped parameter design. For this purpose, they modeled the system with lumped circuit components, such as capacitors and resistors, to calculate the complex impedance in terms of the dielectric constant ($\kappa_{1}$) and the conductivity ($\sigma_{1}$) of the liquid and the applied frequency (ω) [30]. This equivalent lumped circuit model analysis yields a critical frequency ($\omega_{c}$), which determines the predominant mechanism between EWOD and LDEP for the actuation of the aqueous liquid inside the plates. This is due to the fact that the partially conducting liquids behave as perfect conductors and perfect dielectrics below and above the critical frequency, respectively. Jones et al. calculated the frequency-dependent vertical force of electrical origin on the liquid–air interface by integrating the Maxwell stress tensor over a smartly chosen closed surface Σ as shown in Figure 2A [30].

(8) $$F_{z}^{e}={\underset{e}{\to }}_{z}\;\cdot \oint_{\Sigma }^{}{\vec{T}}_{KH}\cdot \vec{n}\;ds={\underset{e}{\to }}_{z}\;\cdot \oint_{\Sigma }^{}\left(\;\varepsilon \vec{E}\vec{E}-\frac{1}{2}E^{2}\vec{I}\right)\cdot \vec{n}\;ds.$$

The closed surface for the integral was chosen in such a way that it must enclose the liquid–air interface, as the LDEP force acts only at the interface; however, the top and bottom faces should be far enough apart to avoid distortions near the corners at the three-phase contact point (liquid, air, and dielectric) due to the fringe electric field, as shown in Figure 2A. With this choice of configuration, the surface integration simply reduces to a summation of the six area contributions from the air denoted by 2, liquid denoted by 5, and dielectrics below and above the air–liquid interface denoted by numbers 1 and 3, and 4 and 6, respectively. The uniform electric fields tangential to the surfaces may be formulated using the resistor/capacitor (RC)-equivalent circuit using the specific capacitances (per unit area) and specific conductance for different components as $C_{d}=\frac{\kappa_{d}\varepsilon_{0}}{d}$, $C_{air}=\frac{\varepsilon_{0}}{d}$, $C_{d}=\frac{\kappa_{1}\varepsilon_{0}}{D}$, and $g_{1}=\frac{\sigma_{1}}{D}$ (see Figure 2B).

The expressions for the electric fields are as follows:

(9) $$E_{d}=\frac{C_{air}}{2C_{air}+C_{d}}V/d;$$

(10) Eair=Cd2Cair+CdVD;

(11) $$E_{d}^{\prime }=Re\left[\frac{j\omega C_{1}}{j\omega \left(2C_{1}+C_{d}\right)+2g_{1}}V/d\right];$$

(12) $$E_{1}=Re\left[\frac{j\omega C_{d}}{j\omega \left(2C_{1}+C_{d}\right)+2g_{1}}V/D\right].$$

The time average of the vertical force of electrical origin can be calculated substituting Equations (9)–(12) in Equation (8) as

(13) $$\langle F_{z}^{e}\rangle =w\left[-\kappa_{d}\varepsilon_{0}E_{d}^{2}d-\frac{\varepsilon_{0}E_{air}^{2}}{2}D+\kappa_{d}\varepsilon_{0}E_{d}^{\prime 2}d+\frac{\kappa_{1}\varepsilon_{0}E_{1}^{2}}{2}D\right].$$

In mechanical equilibrium, the vertical force of electrical origin is equal to the downward pulling gravitational force due to the weight of the liquid column.

(14) $$\langle F_{z}^{e}\rangle =\rho_{1}ghwD.$$

The critical frequency, $\omega_{c}=\frac{{2g}_{1}}{2C_{1}+C_{d}},$ decides the two extreme cases for the liquid actuation. For $\omega \ll \omega_{c}$, $E_{1}\approx 0$, the liquid becomes a perfect conductor, which means that all the voltage drops across the dielectric layer, yielding the actuation of the liquid due to the electrowetting phenomenon. On the other hand, for $\omega \gg \omega_{c}$, the liquid acts as a perfect dielectric as the electric field penetrates into the liquid via polarizing the liquid molecules. In this case, the actuation of the liquid is due to the LDEP force acting as a body force. For the low and high frequency limits, the liquid height may be re-formulated using Equation (14) as

(15) $$h=\{\begin{array}{c} \frac{\kappa_{d}\varepsilon_{0}V^{2}}{4\rho_{1}ghwD}\;\;\;\;\;\;\;\;\;\;\omega \ll \omega_{c}\\ \frac{(\kappa_{1}-1)\varepsilon_{0}V^{2}}{2\rho_{1}gD^{2}}\;\;\;\;\;\;\omega \gg \omega_{c} \end{array}.\;\;$$

Jones et al. also verified these theoretical predictions by measuring the height rise of three different aqueous liquids with varying conductivity (10^−4^–10^−2^ S/m) for a wide range of frequencies using a modified version of Pellat’s experimental set-up [30]. They found a linear relationship for the height rise of the liquids with voltage squared for any given frequency, as predicted by Equation (15). This indicates that either of the mechanisms (EWOD or LDEP) is present for an aqueous liquid when subject to an electric field. However, one of them will be dominant over the other depending on the various conditions such as frequency range, conductivity, dielectric constant of the liquid etc. Therefore, such an RC-equivalent circuit analysis is a very useful and convenient technique to predict the actuation force for the aqueous liquid without knowing the details of the complex fringe electric field at the interface. However, this model lacks the description of how the LDEP force influences the contact angle of the liquid.

### 2.3. Liquid Manipulation in Microfluidics

**Figure 3:**
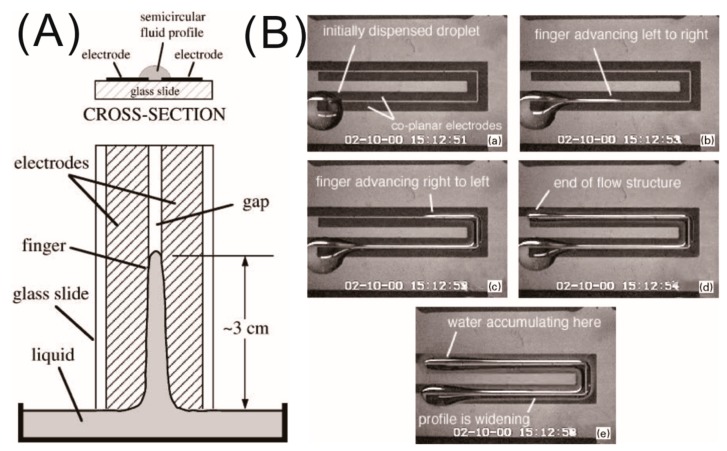
Highly insulating liquid actuation by liquid dielectrophoresis (LDEP) forces using co-planar micro electrodes. (**A**) Cross-sectional view of a dielectric liquid drop sitting on co-planar microelectrodes, and the top view of the spreading of the liquid, stretching like a liquid finger along the gap between the electrodes. (**B**) Sequence of the snapshots of the video micrographs demonstrating water transportation by LDEP force along the patterned electrode gap. Images are adopted from Reference [31] with the permission of Elsevier.

In the early 21st century, with the advent of the advanced microfabrication technique for patterned electrodes in electronics and microfluidics, Jones and co-workers manifested the droplet actuation of μL to nL drops of dielectric liquid on a miniaturized electrode system [17,31]. The electrostatic force generally and especially the LDEP force benefitted from the scaling law, which states that, if the electrode dimension is reduced by a factor α<1, the voltage and the field strength required to maintain a constant DEP force per unit volume scales down $\alpha^{3/2}$ and $\alpha^{1/2}$, respectively [31]. This significantly decreases the voltage requirement of LDEP actuation to very nominal values (200–300 V at 100 kHz). Figure 3A shows the schematic representation of the LDEP experimental set-up with a droplet on top of two co-planar metal electrodes with a width of 1 mm and an electrode gap of 100 μm, patterned on insulating substrates (e.g., SiO_2_ or glass). Upon application of an AC voltage around 200 V root mean square (rms) at 60 Hz, a highly insulating liquid (transformer oil with relative permittivity $\varepsilon_{r}=2.4$) finger was observed to rise to a height of 3 cm, thicker at the base than the top, exhibiting a semi-circular cross-section in the middle as shown in Figure 3A. They also demonstrated the actuation of de-ionized water (DI water), which has electrical conductivity σ in the range of $10^{-4}$ to $10^{-5}$ S/m. To avoid the electrolysis problem associated with conductive liquids, a thin insulating coating of thickness on the order of 10 μm was used to cover the co-planar electrodes. However, this modification in the system led to the use of AC voltage in the radio frequency range, as all the voltage for low frequency only drops across the insulator coating with no polarization effect in the droplet, resulting in no LDEP actuation of the water droplet. Figure 3B shows the series of snapshots of the water transportation via LDEP actuation from a reservoir drop along the patterned electrodes, crossing two 90°-angled corners to reach the other end. They also demonstrated the dispensing of tiny droplets from a mother droplet by breaking the liquid finger through capillary instability at the micro bumps [17]. Later, Prakash et al. described a modified version of the electrode structure with tapered design which allowed more precise dispensing of droplets [32]. Several groups investigated the effect of material properties of electrodes and dielectric coatings, as well as the thickness of dielectric coatings, on the optimized performance of LDEP devices, specifically the actuation voltage and frequency for conducting droplet liquid transport and droplet dispensing operations [19,33,34]. Unlike the co-planar electrode design, Fan et al. used a top and bottom parallel electrode design to produce LDEP actuation for discrete individual droplet manipulation (transportation, splitting, and merging) for digital microfluidics [35]. Several excellent review articles explored LDEP as an actuation technique for microfluidics applications [20,36]. 

## 3. Interface-Localized Liquid Dielectrophoresis

As discussed in the previous sections, liquid dielectrophoresis is used mainly for bulk liquid actuation without a detailed description of the change of shape of a liquid droplet. Similar to electrowetting on dielectrics (EWOD), the wettability of a dielectric liquid drop can be manipulated reversibly if the LDEP force is localized at the solid–liquid interface, using a substrate with an interdigitated electrode (IDE) pattern. Recently demonstrated by McHale et al., this new mechanism for the manipulation of the contact angle of a dielectric liquid drop is called dielectrowetting [21]. In dielectrowetting, the effect of a non-homogeneous electric field is highly localized at the three-phase contact line of the droplet by the interdigitated finger arrangements of electrodes, which are similar to those used in the LDEP phenomenon, in the sense that they are both co-planar. However, such co-planar electrodes were used in electrowetting to manipulate the contact angle of a conducting liquid droplet [12]. In the sections below, the dielectrowetting mechanism and its developments are discussed.

### 3.1. Dielectrowetting

McHale et al. derived the equation for manipulation of the apparent contact angle of dielectric liquid as a function of voltage, which is very similar to the famous Young–Lippmann equation for the electrowetting phenomenon [21]. The energy minimization technique was used to derive the expression, which was verified experimentally overcoming the contact-angle saturation problem faced in EWOD experiments. Dielectrowetting, basically, is the demonstration of the electromechanical force inside the liquid near the solid–liquid interface to produce the spreading of the liquid drop across the substrate. To have a deeper understanding of how the bulk liquid force produces the spreading of the liquid, one needs to comprehend the electric field distribution of the interdigitated electrodes (IDEs). 

**Figure 4:**
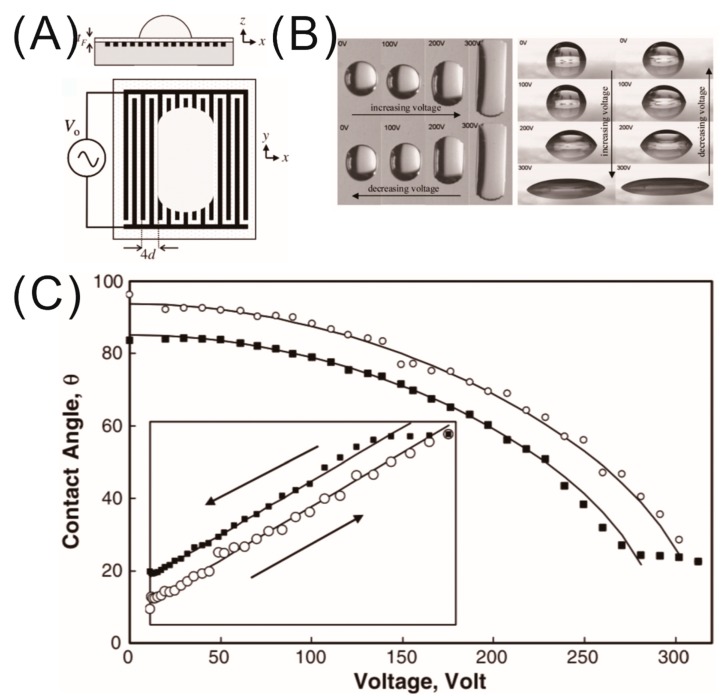
(**A**) Schematic of diletcrowetting experiment (ambient air) depicting the cross-sectional view of a dielectric droplet sitting on a substrate without voltage, and the top view of a spreading droplet on linear interdigitated electrodes (IDEs). (**B**) Optical images of the top and side views of a spreading droplet under different dielectrowetting voltages. (**C**) Apparent contact angle of a stripe-shaped droplet of propylene glycol as a function of applied voltage. Open circles represent the increasing voltage half cycle, and filled squares represent the decreasing voltage half cycle. Inset: linear fit to the cosine of the apparent contact angle versus applied voltage squared. Images are reproduced from Reference [21] with the permission of the American Physical Society.

For dielectrowetting, typically, linear IDEs with the same electrode width and gap d are fabricated on a solid substrate and coated with a thin insulating layer, as shown in Figure 4A. The solution of Poisson’s equation which provides the electrical potential inside the semi-infinite dielectric liquid drop is of the form of V~cos(kx)×exp(−kz), where $k=\frac{\pi }{2d}$ is the wave number [37,38]. Therefore, the electrical potential inside the liquid away from the solid–liquid interface decays as $V\left(z\right)=V_{0}\exp\left(-2z/\delta \right)$, where $\delta =\frac{4d}{\pi }$ is the penetration depth. The penetration depth (δ) is a very important length scale parameter for dielectrowetting as it decides the strength of the penetration of the non-homogeneous electric field into the liquid. By integrating the dielectric energy density $\frac{1}{2}\varepsilon_{0}\varepsilon_{l}{\left|E\right|}^{2}$, where $\varepsilon_{l}$ is the dielectric constant of the liquid and $\vec{E}=-\vec{\nabla }V$, over the volume, the electrostatic energy per unit area stored in the liquid $W_{E}$ can be found as

(16) $$W_{E}=-\frac{\varepsilon_{0}\varepsilon_{l}V_{0}^{2}}{2\delta }\left[\exp\left(-\frac{4h}{\delta }\right)-1\right].$$

In typical experimental conditions, the liquid thickness is very high compared to the penetration depth of the electric field (h≫δ). With this approximation, the electrostatic energy per unit area stored becomes $W_{E}=-\frac{\varepsilon_{0}\varepsilon_{l}V_{0}^{2}}{2\delta }$. This physically signifies that the LDEP is localized within a very small distance inside the liquid of penetration depth (δ) from the solid–liquid interface. This localized force produces a change in contact area of the liquid with the solid to distribute the dielectrophoresis energy within the liquid, yielding a change in apparent contact angle. For a liquid drop sitting on a chemically and topographically homogeneous solid surface, the wetting of the liquid is given by a local minimum in the interfacial energies originating from solid–vapor, solid–liquid, and liquid–vapor interfaces, as described by Equation (1). These are characterized by $\gamma_{SV}$, $\gamma_{SL}$, and $\gamma_{LV}$, representing the energy per unit area. The increase of the contact area by a very small amount, ΔA, means that the solid–vapor interface of the same amount is replaced by the solid–liquid interface and, hence, the increase in the free energy is ($\gamma_{SV}-\gamma_{SL}$) ΔA. In addition, there will be an increase in the free energy of $\gamma_{LV}\Delta A\cos\theta_{e}$ due to the increase in the liquid–vapor interface caused by the liquid movement. The excess electrical energy stored in the extended region caused by the non-uniform electric field due to replacement of the ambient air by the polarized dielectric liquid is $W_{E}=-\frac{\varepsilon_{0}(\varepsilon_{l}-1)V_{0}^{2}}{2\delta }$. The total energy change must vanish for the droplet to attain the equilibrium state with a new value of the contact angle $\theta_{V}$, given by

(17) $$\cos\theta_{V}=\cos\theta_{e}+\frac{\varepsilon_{0}(\varepsilon_{l}-1)V_{0}^{2}}{2\gamma_{LV}\delta },$$

where $\theta_{e}$ is Young’s contact angle. Equation (17) predicts the apparent contact angle change of a dielectric liquid as a function of applied voltage similar to the electrowetting equation; however, the ratio of the dielectric constant of the insulator coating to its thickness ($\frac{\varepsilon_{r}}{d}$) in the later one is replaced by the ratio of the liquid dielectric constant minus the dielectric constant of the medium to the electric field penetration depth $\frac{\left(\varepsilon_{l}-1\right)}{\delta }$. 

In the same paper, McHale et al. experimentally confirmed the relationship between the contact angle and applied voltage using a polypropylene droplet on a substrate with linear IDE as shown in Figure 4 [21]. A hydrophobic thin top coating was employed to start the experiment with a high initial contact angle for large modulation of the contact angle. As shown in Figure 4C, the apparent contact angle of the droplet exhibited a large modulation of around 70° upon application of a voltage of 300 V, overcoming the contact-angle saturation limit usually observed in EWOD experiments. However, a small hysteresis between the increasing and decreasing voltage cycle was observed. This irreversibility of the apparent contact angle arises from the pinning of three-phase contact line at the surface roughness, which was resolved later by Brabcova et al. by infusing a lubricant in the roughness of the substrate [39]. The inset of Figure 4C shows a clear linear dependence of the cosine of the apparent contact angle with the square of the applied voltage $V_{0}^{2}$, which confirms the relationship of Equation (17). Moreover, Equation (17) predicts that, for a sufficiently high threshold voltage ($V_{th}$), the droplet could be spread to a thin film with zero contact angle. With this threshold voltage $(V_{th}$), the dielectrowetting behavior can be predicted by the following model [23]:

(18) $$\cos\theta_{V}=\cos\theta_{0}+\left(1-\cos\theta_{0}\right){\left(\frac{V_{0}}{V_{th}}\right)}^{2},$$

where $\theta_{0}$ is the contact angle of the droplet at zero applied voltage, and $V_{th}$ is the threshold voltage when $\theta_{V}=0{}^{\circ}$, i.e., complete wetting is predicted. However, Equation (10) is only valid for h≫δ; as a result, the zero contact angle could never be achieved in reality, as, for a spread thin film, the height of the drop approaches the penetration depth. For h≪δ, the electric field becomes very strong such that it starts to deform the liquid–vapor interface, giving rise to the surface wrinkling [37,38]. 

### 3.2. Wetting Manipulation in Two-Phase Liquids

In two-liquid systems, the contact angle of the droplet is, typically, determined by the interfacial tension of the liquid–liquid (medium). The liquid medium plays a very important role in LDEP-based wettability manipulation, as, contrary to the case in air medium, the contact angle could increase upon the application of voltage if the ambient has higher polarizability than the droplet liquid, i.e., $\left(\varepsilon_{m}>\varepsilon_{l}\right)$. Yang et al. demonstrated manipulation of the contact angle of a dielectric liquid drop in a highly polarizable liquid medium through an interface-localized LDEP force using concentric interdigitated electrodes with an electrode width and gap of 30 μm and 10 μm, respectively [40]. The largest apparent contact angle around 176° with an applied voltage of 215 V was achieved for a silicone oil ($\varepsilon_{l}=2$) droplet with an initial contact angle 20° inside polyalcohol ($\varepsilon_{m}=45$). However, the change of contact angle does not follow the usual quadratic relationship describe by Equation (17) at higher voltages. This deviation could be due to many factors: the non-linearity in the electrode design, buoyancy force effect, and transition of the shape of the droplet from a spherical cap to an almost spherical ball with considerably less of a liquid–liquid interface near the electrode. This fundamental issue is not yet understood well and could be a problem to be resolved for better applicability of the reverse wetting manipulation by the LDEP technique in two-fluid microfluidic systems. Brown et al. studied the dielectrowetting droplet spreading in another immiscible liquid with lower dielectric constant [24]. They also developed a theoretical model by considering the droplet shape as a rectangular cuboid of constant volume Ω that predicts the decrease of the height of the liquid droplet with the square of dielectrowetting voltage as follows [24]:

(19) $$h^{2}\left(V\right)=h_{0}^{2}-\frac{\varepsilon_{0}\left(\varepsilon_{l}-\varepsilon_{m}\right)\Omega }{4\delta \gamma_{lm}}V_{0}^{2}.$$

Equation (19) was experimentally verified using droplets of six different test liquids in six different liquid media. They also showed that dielectrowetting in the liquid–liquid system could be an effective method of determining material properties such as interfacial tension or permittivity from the ratio of $\frac{\Delta \varepsilon }{\gamma_{lm}}$.

### 3.3. Anisotropic Spreading

In dielectrowetting, the spreading direction of the dielectric liquid drop is dictated by the geometry of the interdigitated electrodes, as the drop prefers to spread in the parallel direction of the electrodes, resulting in huge modulation of apparent contact angle along one direction only [21]. The liquid experiences hindrance in spreading along the perpendicular direction of the IDEs, as the periodic variation of electric potential acts as a barrier to the spreading. However, in the dielectrowetting experiments by Geng et al., the spreading in the perpendicular direction of the drop overcoming the periodic potential barrier was observed at high applied voltage due to the blocking of the spreading of the drop in the parallel direction at the edge of the electrodes [41]. This potential barrier could be thought of as virtual inhomogeneity, analogous to the physical roughness and chemical inhomogeneity present on real surfaces, which usually hinders the movement of the three-phase contact line of the drop during the spreading driven by dielectrowetting. Mannetje et al. demonstrated that the drop motion could easily be controlled, and even trapped at the virtual defect engineered by the electrical potential [42]. Therefore, the periodic potential barriers originating from the interdigitated electrodes could be used as a model experimental system to study the origin of contact-angle hysteresis and the dynamics of the three-phase contact line under an inhomogeneous electric field. A better system for the study of the pinning phenomenon would be the concentric electrode design demonstrated by Cheng et al. for a liquid dielectric lens [43]. The liquid drop takes a spherical cap shape at equilibrium with the quantization of contact angle due to the periodicity of the electric field, acting as the virtual pinning sites in the radial direction. However, the discreteness in spreading for concentric electrodes and the non-axis symmetric spreading of the linear interdigitated electrodes could be a major problem for the application of dielectrowetting, as many applications require specific axis-symmetric and smooth manipulation of the contact angle. To solve the problem of the periodic potential barrier, Russel et al. engineered electrodes with a special “zippered” design, which had electric field components in both directions [44]. This special design helped the drop spread in slightly axis symmetry. However, the spreading was limited by the longer supplying electrodes in the parallel direction. Russel et al. demonstrated an improved version of the electrode design with 500 nm of Paralene C with 50 nm of FluoroPel 1601 V dielectric stacking, showing omni-directional spreading of the drop [45]. Brabcova et al. reported smooth spreading of a droplet on a substrate with circular electrode geometry, driven by AC signals having phase shifts of 90° (0°, 90°, 180°, and 270°) [46]. Another alternative technique to manipulate the contact angle axis-symmetrically and smoothly was demonstrated by Xu et al. using radial interdigitated electrodes, having a gradient in electrode gap from the center to the outward direction [47]. By increasing the number of electrodes, the authors reported an increase in the dielectrophoresis force to have higher manipulation with the same applied voltage.

## 4. Recent Applications 

### 4.1. Digital Microfluidics Applications 

**Figure 5:**
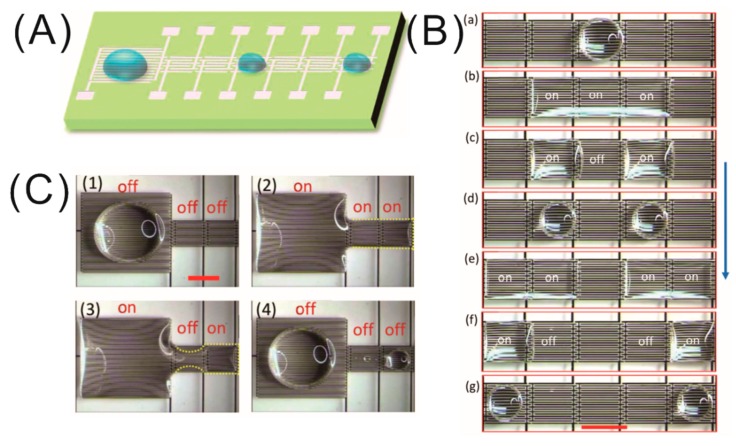
(**A**) Schematic representation of the experimental open microfluidics device with a large IDE pad for a reservoir and smaller IDE pads for various microfluidics operations. (**B**) Series of snapshots explaining the droplet splitting (**a**–**d**) and transporting (**e**–**g**) operations. Scale bars are 2 mm. (**C**) Droplet generation on the device by dielectrowetting. Scale bars are 2 mm. Images are reproduced from Reference [41] with the permission of the Royal Society of Chemistry.

With the huge advancement in science and technology in the 21st century, a natural trend in modern technology is to manufacture miniaturized, cheap, and easy-to-handle devices. The emerging microfabrication technology made it possible to integrate many biological and chemical analysis processes into a miniaturized chip, known as the micro total analysis system (μTAS) or lab-on-a-chip (LoC) technology. In recent decades, the modern medical diagnostic, bio technology, and microfluidic research greatly benefited from these technologies due to their various advantages, such as portability, versatility, high speed, and high throughput with extremely small volumes of samples and reagents [48,49,50,51]. The control of microfluids is a very essential task inside an LoC system and can be achieved mainly in two ways; one involves channel-based continuous microfluidics, and the other involves digital microfluidics. In digital microfluidics, the basic four droplet operations (creating, transporting, splitting, and merging) are routinely achieved by the EWOD technique in microfluidic systems consisting of the two parallel plate configuration. On the other hand, the development of the LDEP technique emerged as an alternative driving mechanism for microfluidics, as discussed in the section on LDEP in microscale. Many excellent reviews exploring the wide field of continuous and digital microfluidics were published in the literature [52,53]. Here, we discuss the application of the dielectrowetting technique in the devices. Recently, a critical and thorough review on digital microfluidics based on dielectrowetting was covered by Geng and Cho [54]. The experimental realization of dielectrowetting as the driving mechanism in digital microfluidics using interdigitated electrodes was demonstrated by Geng et al. [41] The authors demonstrated the basic four droplet operations, i.e., creating, transporting, splitting, and merging, on open microfluidic platforms consisting of a combination of linear IDEs with one larger area (5 mm × 5 mm) and six smaller working areas of 2 mm × 2 mm, as shown in Figure 5A [41]. Upon the application of a high AC signal (~20 kHz), the dielectric liquid (propylene carbonate,) spreads over many addressable electrodes at the same time, forming a stretched thin film due to a strong interface-localized LDEP force inside the dielectric fluid. Once the droplet was stretched over three activated electrodes, the splitting of the droplet was executed by switching off the middle electrode, which changes the energy of the liquid, and, as a result, the liquid prefers to stay on the activated electrodes, leaving the non-activated one, thus creating two droplets (Figure 5B). Later, the electrical signal was withdrawn from the electrodes to complete the splitting process to get two half-spherical droplets. Using the similar operation principle of droplet splitting, the authors could also generate droplets with desired volumes from a bigger droplet initially sitting on the reservoir electrode. The droplet merging was achieved by turning on the middle electrode pad and later turning off all three activated electrodes to achieve a half-spherical droplet in equilibrium. Next, the transporting of the droplet was a slightly different process than the previous two. Firstly, the droplet was stretched over adjacent electrodes by turning on the electrical signal, followed by switching off the electrode on which the droplet initially resides, as shown in Figure 5B (e–g). The most important aspect of the dielectrowetting technique is that all the operations were achieved in an open environment without requiring a top electrode plate, which is an essential part of the electrode system for the EWOD-based microfluidics operations. All the biomedical applications and biological research require a conducting liquid, commonly aqueous solutions, as the working medium for operations in microfluidic devices. In this study, very interestingly, the authors demonstrated the microfluidic operations not only for a dielectric liquid (i.e., propylene carbonate) but also for conducting liquids (DI water with and without surfactant), employing a higher-frequency (~55 kHz) AC electrical signal to push the limit of an open digital microfluidic system. This study paved the way for interesting and advanced future microfluidic systems with the flexibility of liquid choice as per the requirement of the system and increased functionality.

### 4.2. Dielectrowetting-Based Liquid Lens

A classical spherical lens, typically made from solid materials such as glass or plastic material, refracts the light passing the interface depending on the contrast of the refractive indices of the media. Although solid lenses are highly scalable in size, they lack the flexibility to adapt the variable focal lengths. On the contrary, liquid lenses are highly flexible, as the focal length can be tuned by two mechanisms; one involves controlling the spatial distribution of the refractive index of the anisotropic optical medium (nematic liquid crystals) and another involves tuning the curvature of the liquid–vapor interface of a droplet. The controlled tuning of the focal length of a liquid crystal (LC) lens can be achieved by a non-uniform electric field via spatial distribution of the refractive index in the nematic LCs [55,56,57,58,59]. The nematic LC molecules are essential in the LC lens due to its anisotropy in terms of optical properties and dielectric constant. The LC molecular axis with a higher dielectric constant orients parallel to the applied external electric field due to the electrostatic torque on the molecules that induces the lensing effect with adjustable focal length. A recent paper by Lin et al. thoroughly reviewed the literature on the tunable liquid crystal lens [60]. 

The electrical tuning of focal length was also demonstrated by electrowetting and dielectric force via controlled deformation of the interface profile of a droplet [61]. Obviously, the contact-angle manipulation of a sessile droplet by the dielectrowetting technique provides a better alternative way to achieve liquid lenses than the EWOD technique, due to the fact that a variety of liquids with different dielectric constants and optical properties can be actuated in the former technique. In 2006, Cheng et al. were the first to demonstrate a dielectrophoresis-based liquid lens via tuning the curvature of a liquid crystal droplet on concentric interdigitated electrodes with a spacing of 50 μm [43]. They achieved focal length variation between 1.6 mm and 2.6 mm without and with application of a voltage of 240 V at 1 kHz, respectively. The consumed power was about 0.1 mW for a numerical aperture of 0.5 mm with a very fast response time of 220 ms. This work was followed by Cheng and Yeh, where the authors demonstrated a package of liquid lens consisting of a low-dielectric-constant liquid droplet and a high-dielectric-constant sealing liquid environment driven by the dielectric force [62]. The mass density of the sealing liquid was chosen in such a way that it matched the droplet liquid to avoid the optical aberrations due to the deformation of the droplet induced by the gravitational force. Upon the application of an AC signal of 200 V at 1 kHz, the focal length of the liquid lens with an aperture of 3 mm was changed from 34 mm without voltage to 12 mm with the maximum power consumption of 1 mW. However, the rise and fall time of the lens significantly increased to 650 ms and 300 ms, respectively due to the viscous dissipation effect of the ambient liquid. Since these pioneering studies, there were many efforts by other groups to reduce the power consumption for the operation of liquid lenses by creatively designing electrodes with a lower actuation voltage [47,63]. Zhang et al. experimentally verified the influence of temperature on the two-liquid dielectric liquid lens, which displayed an exponential variation of focal length over a threshold temperature [64]. This increase is due to the combinational change in both the refractive index of the liquid and the liquid–vapor interface with elevated temperature while the former changes linearly. The lens has the potential to fully recover its initial properties and focal length even after heating up to 130 °C. Another important aspect is the flexibility of the liquid lens system, which could add the advantage of reconfigurability of the system. Recently, Lu et al. fabricated a flexible dielectrowetting-based liquid lens by pattering concentric electrodes embedded in a polydimethylsiloxane (PDMS) substrate [65].

### 4.3. Dielectric Switch and Display Applications

**Figure 6:**
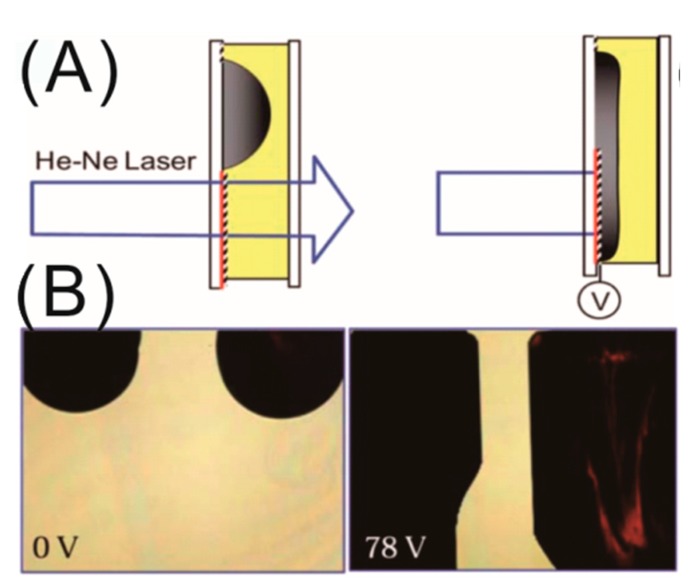
(**A**) Schematic representation of the working principle of the optical switch based on dielectrowetting spreading of liquid drop. (**B**) Liquid crystal droplet-based optical switches in the off and on (78 V) state. The left droplet shows a black color and the right one shows red. Images are reproduced from Reference [66] with permission of the Royal Society of Chemistry.

In 2011, the first optical switch based on the dielectrowetting mechanism was developed by Ren et al. [66]. The authors demonstrated the operation of the optical switch by blocking an He–Ne laser light with the help of a spreading liquid crystal droplet doped with 1.2 wt.% black dye via the dielectrowetting force, employing linear interdigitated electrodes (see Figure 6A), while the sideways spreading of the droplet was achieved using a zipper electrode design. However, the switch operating time (i.e., spreading time ~0.39 s and recovery time ~0.75 s) was relatively slower, which confirms the extreme spreading of the droplet. In the transmission mode, the switching contrast ratio was as high as 120:1. Later, the same group demonstrated the operation of a single-pixel dielectrowetting-based display which can operate in both reflective and transmissive modes [67]. The authors also demonstrated a simple 1 × 6 color pixel array and the improvement in the contrast ratio of the pixels by changing the dye concentration; however, that resulted in a poor transmittance performance. Zhao et al. demonstrated an optical shutter based on liquid film break-up by the dielectrowetting mechanism using interdigitated electrodes [68]. In the voltage off state, the optical shutter exhibited a very high light transmission of ~80%. Luo et al. developed a dielectrowetting display with enhanced color performance using a patterned quantum dot (QD) array [69]. The application of QDs benefited the system with outstanding color gamut (136% AdobeRGB in CIE1976 color space) with a huge reduction in the optical loss in each color filter by pre-converting the light to desired colors. They also improved the energy efficiency while maintaining the high backlight transmission and fast response time. Recently, Wang et al. scaled up the display area using an array of miniaturized pixels of 1.0 mm × 0.5 mm with a polymeric wall of 50 µm separating each one, preventing the droplets on each pixel from coalescing during the spreading operation [70]. The authors also improved the response time of the switches, exhibiting faster switching with increasing voltage. These early demonstrations of the optical switch systems with expected further improvements in the future could potentially lead to the development of dielectrowetting-based displays for e-paper and mobile phone applications.

### 4.4. Miscellaneous Applications

In recent years, dielectrowetting gained huge popularity as an active and alternative tool for manipulation of the wetting property of a non-conducting droplet, which is reflected in the modern literature, as scientists came up with various ideas to explore the technique in novel applications in real life. Recently, a couple of research groups demonstrated a liquid iris based on the dielectrowetting phenomenon [71,72]. Tsai and Yeh first built a liquid iris which was composed of two nonconductive immiscible liquids (silicone oil and a propyl alcholol with an opaque ink) on a glass substrate with a set of driving concentric electrodes [71]. This iris had the capability to vary its aperture from 4 mm without voltage to 1.5 mm at 160 V rms with a maximum electric power consumption of 5.7 mW. Later, Xu et al. demonstrated a better electrode design for their liquid iris system which enabled achieving a 94% aperture ratio with the application of an actuation voltage of 70 V rms [72]. Wells et al. demonstrated the fabrication of a diffraction grating with a suppressed zeroth-order diffraction pattern, using the dielectrowetting force as a means to imprint a periodic corrugation with a pitch of 20 µm in the final shape of the surface of a uniform film of UV-curable resin liquid [73]. Similarly, several research groups exploited the dielectrowetting method with simultaneous UV treatment or heating as per the requirement of the corresponding responsive polymers for curing in the micro-fabrication of the microfluidics devices and mciro-lens array systems [74,75]. The benefit of this technique is that there is no requirement of the photo-lithographic mask for a polymer master mold, which hugely reduces the time consumption and labor for the fabrication of such prototype new devices. Dielectrowetting was also used to studying and controlling different physical fundamental phenomena such as the study of the dewetting phenomenon of a liquid film into a droplet and the active control of the Cheerios effect [76,77,78]. Edwards et al. recently showed that the de-wetting process of a liquid film into a droplet, which is purely driven by capillary forces, is not the time reversal process of the spreading of a droplet [76]. However, achieving a completely liquid film which would de-wet spontaneously was a very challenging task, which was possible upon the application of dielectrowetting force on a dielectric liquid drop to super spread using circular concentric electrodes. Another interesting study is the dielectrowetting-based active control of the Cheerios effect, commonly observed among floating objects on a liquid which are attracted or repulsed by each other and the side wall, performed by Yuan et al. [77,78]. The modulation of the attraction and the repulsion between the particle and the side wall was achieved via manipulating the contact angle of the liquid through the dielectrowetting force on an interface localized on the interdigitated electrodes on a side wall with 10° tilt. Later, the effect was utilized to demonstrate the propulsion of floating particles through a small channel by switching the electrode pairs from either side of the channels. This study widens the potential possibility for future applications in self-assembled monolayers of particles at the liquid interfaces on a large scale via manipulating the attraction or repulsion behavior of particles.

## 5. Conclusions and Outlook

This paper provided a brief overview of the LDEP phenomenon for liquid manipulation and its latest version of the dielectrowetting phenomenon, in which the contact angle of a dielectric liquid droplet can be modified reversibly though the localization of the LDEP force at the solid–liquid interface. LDEP is a bulk force of electrical origin which penetrates into the bulk of the dielectric liquid through the polarization of liquid molecules to produce a movement toward the higher-intensity direction of a non-homogeneous electric field. The RC-equivalent circuit analysis of the LDEP system predicts that the actuation of aqueous liquids by the LDEP force using high frequency, much higher than the critical frequency, can be achieved. The fundamental concept of dielectrowetting is the reversible modification of the apparent contact angle through localizing the LDEP force at the three-phase contact line to deform the liquid–vapor interface. Apparently, the contact-angle change behavior has similarity with the electrowetting on dielectrics, but the electrode design (co-planar linear interdigitated electrodes instead of planar electrodes) and the basic mechanism are very different from the latter. The main advantage of dielectrowetting is that a dielectric liquid drop can easily be spread into a thin film at sufficiently high voltage, overcoming the limitation of the contact-angle saturation encountered in EWOD. With the capability of super spreading a dielectric liquid drop, dielectrowetting was applied to microfluidics and optofluidics devices. 

Although dielectrowetting is in its early stage, many problems such as axis-asymmetric spreading of the liquid droplet with an advanced engineered electrode pattern and contact-angle hysteresis using a slippery surface were solved. However, the actuation voltage for contact-angle manipulation by dielectrowetting is quite high compared to the EWOD technique, which restricts it from application in devices with a low power requirement. This issue could be solved by the combination of better electrode design and a high-dielectric-constant liquid. One possible future research direction for the principle of dielectrowetting could be the investigation of the physics of contact line pinning during spreading at virtual pinning sites created by the periodic potential in the perpendicular direction of the linear interdigitated electrodes.
